# Metabolomic Study of High-Fat Diet-Induced Obese (DIO) and DIO Plus CCl_4_-Induced NASH Mice and the Effect of Obeticholic Acid

**DOI:** 10.3390/metabo11060374

**Published:** 2021-06-10

**Authors:** Nanlin Zhu, Suling Huang, Qingli Zhang, Zhuohui Zhao, Hui Qu, Mengmeng Ning, Ying Leng, Jia Liu

**Affiliations:** 1 Shanghai Institute of Materia Medica, Chinese Academy of Sciences, Shanghai 201203, China; zhunanlin@simm.ac.cn (N.Z.); qingli.zhang@simm.ac.cn (Q.Z.); 2 State Key Laboratory of Drug Research, Shanghai Institute of Materia Medica, Chinese Academy of Sciences, Shanghai 201203, China; slhuang@simm.ac.cn (S.H.); 201628012342080@simm.ac.cn (Z.Z.); quhui@simm.ac.cn (H.Q.); ningmengmeng@simm.ac.cn (M.N.); 3 University of Chinese Academy of Sciences, Beijing 100049, China

**Keywords:** nonalcoholic fatty liver disease (NAFLD), nonalcoholic fatty liver (NAFL), nonalcoholic steatohepatitis (NASH), differential metabolites, metabolic pathways, obeticholic acid (OCA)

## Abstract

The pathophysiology of nonalcoholic fatty liver disease (NAFLD) is a complex process involving metabolic and inflammatory changes in livers and other organs, but the pathogenesis is still not well clarified. Two mouse models were established to study metabolic alteration of nonalcoholic fatty liver and nonalcoholic steatohepatitis, respectively. The concentrations of metabolites in serum, liver and intestine content were measured by the AbsoluteIDQ^®^ p180 Kit (Biocrates Life Sciences, Innsbruck, Austria). Multivariate statistical methods, pathway analysis, enrichment analysis and correlation analysis were performed to analyze metabolomic data. The metabolic characteristics of liver, serum and intestine content could be distinctly distinguished from each group, indicating the occurrence of metabolic disturbance. Among them, metabolic alteration of liver and intestine content was more significant. Based on the metabolic data of liver, 19 differential metabolites were discovered between DIO and control, 12 between DIO-CCl_4_ and DIO, and 47 between DIO-CCl_4_ and normal. These metabolites were mainly associated with aminoacyl-tRNA biosynthesis, nitrogen metabolism, lipid metabolism, glyoxylate and dicarboxylate metabolism, and amino metabolism. Further study revealed that the intervention of obeticholic acid (OCA) could partly reverse the damage of CCl_4_. The correlation analysis of metabolite levels and clinical parameters showed that phosphatidylcholines were negatively associated with serum alanine aminotransferase, aspartate aminotransferase, NAFLD activity score, and fibrosis score, while lysophosphatidylcholines, sphingomyelins, amino acids, and acylcarnitines shared the reverse pattern. Our study investigated metabolic alteration among control, NAFLD model, and OCA treatment groups, providing preclinical information to understand the mechanism of NAFLD and amelioration of OCA.

## 1. Introduction

Nonalcoholic fatty liver disease (NAFLD), characterized by excessive cytoplasmic retention of triglyceride, is one of the most common form of chronic liver disease [1,2]. It encompasses a spectrum of conditions ranging from non-progressive nonalcoholic fatty liver (NAFL) to progressive nonalcoholic steatohepatitis (NASH) and is strongly associated with metabolic syndrome (obesity, type 2 diabetes mellitus, dyslipidaemia and hypertension) [3,4]. However, the mechanisms of the development of simple steatosis (NAFL) and the following transition to steatosis, inflammation, ballooning, and/or fibrosis (NASH) are still not well understood. A previous human study has revealed that the gut epithelial permeability was increased in NAFLD patients, led to a higher exposure of intestine-derived bacterial products such as fatty acids, LPS, bile acids to liver, which would exacerbate the hepatic inflammation and dyslipidaemia [5]. To further understand the metabolic and inflammatory changes of the involved multi-organ in NAFLD pathogenesis, we explore the metabolites in serum, liver and intestine content of control, high-fat diet (HFD)-fed obese (DIO) mice, HFD plus carbon tetrachloride treatment (DIO-CCl_4_) mice, and performed correlation analysis among metabolites, hepatic histology characterization and other biochemistry makers. Moreover, OCA, a farnesoid X-receptor (FXR) agonist finished with Phase III clinical trials was administered to DIO-CCl_4_ mice, and correlation analysis among metabolites and the OCA-mediated amelioration in NASH marker was performed. Preclinical information was provided in the present study and might be helpful to understand the mechanism of NAFLD and drug modulation.

Metabolites, as the downstream products of other biomolecules (such as genes, mRNAs and proteins), actively interact with all levels of biomolecules, thus play essential and crucial roles in biological systems [6,7,8,9]. Based on the concentrations and variations of metabolites, systematic metabolic changes caused by internal or external disturbances such as diet, environment, drug and genetic effects could be directly learned [8,10,11]. Moreover, metabolite-driven phenotype modulation has been discovered, such as modulation of neuronal excitability and plasticity by lactate [12], modulation of macrophage activation and regulation of immunity by α-ketoglutarate [13], and taurine-induced myelin basic protein expression [14]. Metabolomics specializes in the analysis of metabolites; hence it is considered as a useful approach to understand the pathogenesis and related mechanisms of NASH.

In addition, a lot of scientists have paid substantial efforts in drug development of NAFLD. Nowadays, several drugs were on clinical trials. Obeticholic acid (OCA), as the first-in-class of FXR ligands, has been approved in May 2016 for the treatment of primary biliary cholangitis (PBC). And the current studies are still ongoing for its potential in the treatment of NASH [15]. However, the mechanisms by which OCA can attenuate metabolically induced inflammation and associated pathways leading to NASH remain not well understood, and the investigation of metabolomics is deficient. Our research anticipated to provide metabolic clues to understand the mechanism of the treatment of OCA.

In this study, we established NAFLD models (DIO group as NAFL-like model and DIO-CCl_4_ group as NASH-like model) in mice. Then we applied a target metabolomic approach to analyze metabolic alteration of liver, serum and intestine content. The differential metabolites among NAFLD models and control were discovered by multivariate (MVA) statistical methods, and the disturb pathway was also analyzed. In addition, the correlation analysis was also performed to give insight into association between differential metabolites (among NAFLD models and control group) and clinical parameters. Depending on the metabolic results of liver, the correlation with modulated effect of OCA for the treatment of NASH was also investigated.

## 2. Results

### 2.1. The Histological Characteristics of NAFLD Models and OCA Group

**Figure 1:**
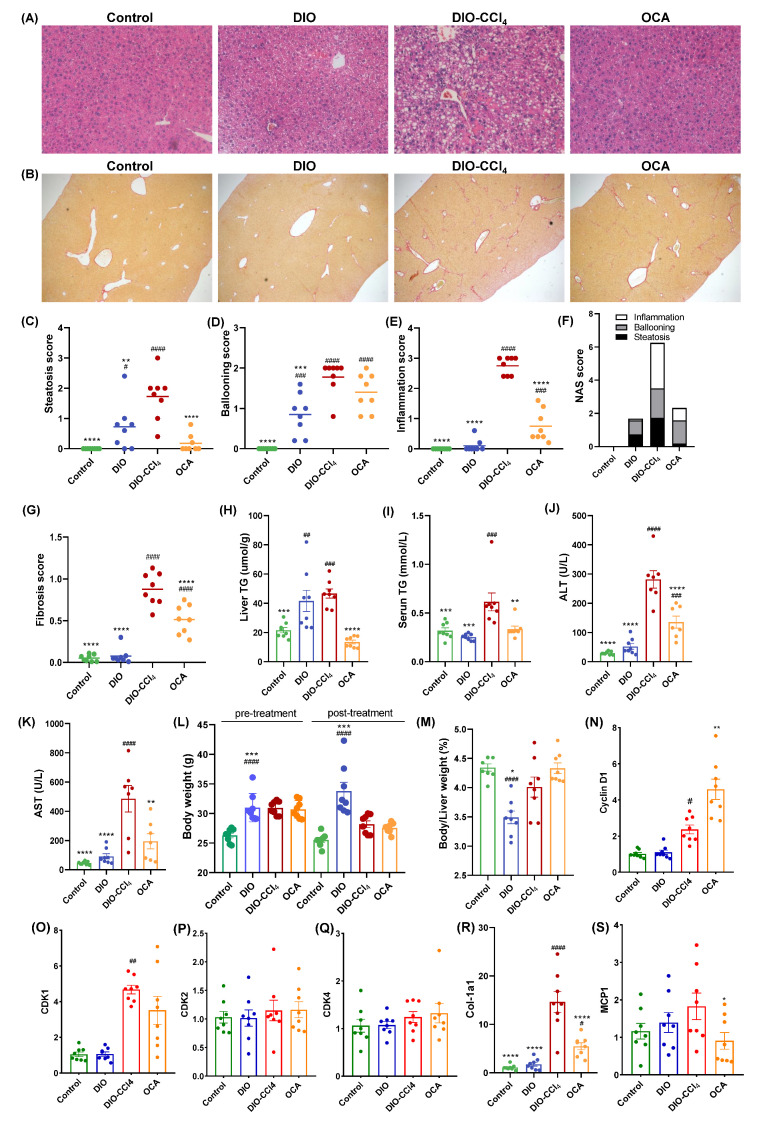
OCA attenuates hepatic steatosis, inflammatory responses and fibrosis in DIO-CCl_4_ mice. (**A**) Representative Hematoxylin and eosin (HE) staining results (magnification ×200). (**B**) Representative Sirius red staining results (magnification ×50). (**C**–**F**) Histological assessment of steatosis score, ballooning score, inflammation score and the NAFLD activity score (NAS). (**G**) Quantification of Sirius Red-positive area. (**H**) Liver TG was detected. (**I**–**K**) The serum TG concentration, ALT and AST was measured. (**L**) Body weight and (**M**) liver/body ratio. (**N**–**S**) Gene expression assessment of cyclin D1, CDK1, CDK2, CDK4, Col-1a1 and MCP-1 in livers. * *p* < 0.05, ** *p* < 0.01, *** *p* < 0.005 and **** *p* < 0.001 vs. DIO-CCl_4_ group; ^#^ *p* < 0.05, ^##^ *p* < 0.01, ^###^ *p* < 0.005 and ^####^ *p* < 0.001 vs. Control group.

The high-fat diet (HFD) fed obese mice, exhibiting fatty liver but not steatohepatitis, were established as NAFL-like murine model [16]. In accordance with the literature, HFD-induced obesity supplemented with multiple administration of CCl_4_ is sufficient to establish a NASH-like murine model that mimics several features of human NASH [16]. As shown in Figure 1A, the results of the histological examination, using H&E staining, revealed that HFD induced steatosis in the livers of DIO group. Consistent with previous reports, co-treatment with CCl_4_ caused a further hepatocyte ballooning, inflammation, and moderate peri-sinusoidal or peri-portal fibrosis in the livers of DIO-CCl_4_ group (Figure 1A–G), and significant increases in serum TG, AST, and ALT were observed after CCl_4_ treatment in DIO mice, when compared with control mice (Figure 1I–K). Accordingly, the hepatic TG level and Col-1a1 expression significantly increased in both DIO and DIO-CCl_4_ groups compared to the control group (Figure 1H,R). Histological characterization of OCA group was performed, indicating that the treatment of OCA could significantly reduce the steatosis score and liver inflammation score by 89.9% and 72.7%, respectively, compared with vehicle group (Figure 1C,E). Among them, steatosis score showed no significance to that of control group. Treatment with OCA reduced serum ALT by 50.9% and serum AST by 62.9% against the vehicle control (DIO-CCl_4_). OCA reduced the serum triglycerides l by 45.4% but showed no obvious effect on serum cholesterol. The NAS score was also significantly reduced after OCA treatment, with a 62.8% reduction (Figure 1F), though the ballooning score was almost unchanged. Treatment with OCA reduced serum triglycerides, ALT and AST by 45.4%, 50.9% and 62.9% against the vehicle control (DIO-CCl_4_), respectively (Figure 1I–K). The liver/body weight ratio (%) of OCA was 8% higher than the DIO-CCl_4_ group but did not reach statistically significance (Figure 1M). As FXR agonists are known to activate proliferation pathways [17], the impact of OCA on gene expression of cyclin D1, CDK1, CDK2 and CDK4 were investigated. CCl_4_ treatment caused an increase of cyclin D1 and CDK1 gene expression (Figure 1N,O), while the expression of CDK2 and CDK4 were unaffected (Figure 1P,Q). OCA treatment caused a further increase of cyclin D1. However, the expression of CDK1, CDK2 and CDK4 were unaffected compared to DIO-CCl_4_ group (Figure 1N–Q). The positive areas of Sirius red staining were reduced by 41.5% after OCA treatment compared with DIO-CCl_4_ group (Figure 1G), indicating an amelioration in fibrosis. Accordingly, the expression of Col-1a1, a marker of the fibroblast-like phenotype, was increased in DIO-CCl_4_ group and significantly inhibited by OCA treatment (Figure 1R). Consistent with the lowered inflammation score, the expression of chemokine monocyte chemoattractant protein-1 (MCP-1) in liver was significantly decreased by OCA treatment (Figure 1S).

### 2.2. Metabolic Characteristics of NAFLD Models and Control Group

**Figure 2:**
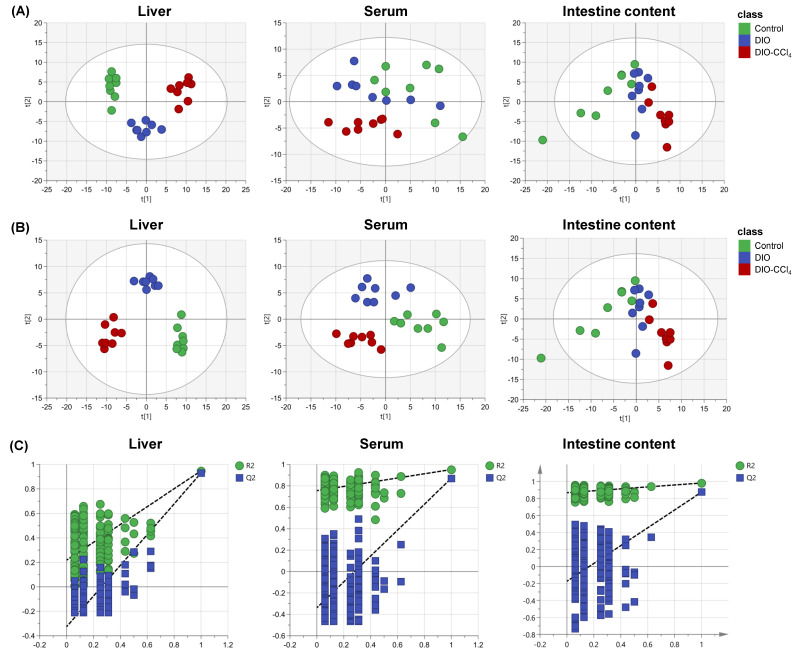
The metabolomic analysis of liver, serum and intestine content. (**A**) PCA score plots of liver, serum and intestine content. (**B**) PLS-DA score plots of liver, serum and intestine content. (**C**) Model validation using a permutation test with 200 cycles of liver, serum and intestine content.

**Figure 3:**
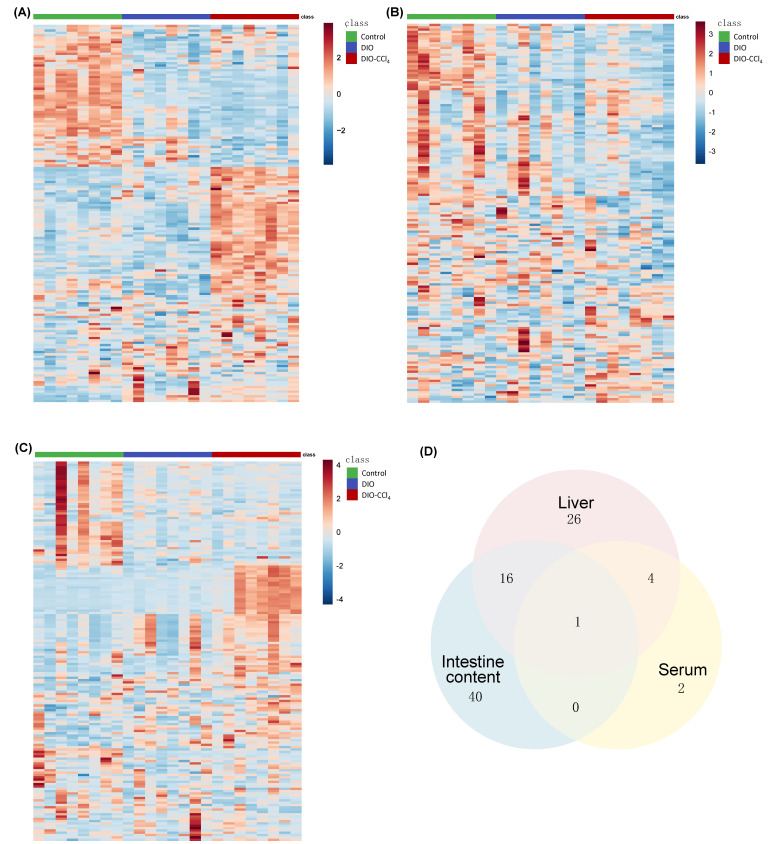
The metabolomic analysis of liver, serum and intestine content. Heatmaps of metabolic characteristics of liver (**A**), serum (**B**) and intestine content (**C**). (**D**) Venn plot of differential metabolites (The number means the shared differential metabolites).

To obtain the metabolic characteristics of liver, serum and intestine content samples, target metabolomic approach was carried out using the AbsoluteIDQ^®^ p180 Kit (Biocrates Life Sciences, Innsbruck, Austria) which could measure the concentration of 188 metabolites accurately. Multivariate (MVA) statistical analysis was conducted to observe whether the metabolic disturbance was occurred in the NASH models. Firstly, PCA, as a frequent unsupervised model, was established based on the corresponding data (Figure 2A). The results showed that the samples could be well grouped and separated by different groups in liver, serum and intestine content samples. And the aggregation and separation of liver was the best, comparing to serum and intestine content samples. The farthest distance was observed between DIO-CCl_4_ group and control group in PCA plots, while DIO group was closer to the control. These results further indicated that the disorder in the DIO-CCl_4_ group might be more significant. And then, a supervised PLS-DA was applied to detect an obvious separation shift among control and NASH model groups (Figure 2B). A clearer discrimination of each group was observed in the PLS-DA score plots. The R^2^Y and Q^2^ values indicated good explanation ability and predictive capacity of the model (R^2^Y intercept of liver, serum and intestine content was 0.925, 0.954 and 0.979, while the Q^2^ intercept was 0.892, 0.850 and 0.861, respectively). A permutation test with 200 cycles was used for checking whether the model was overfitting (Figure 2C). And the intercepts of R^2^ and Q^2^ values suggested that no overfitting was found (R^2^ intercept of liver, serum and intestine content was 0.219, 0.754 and 0.867, while the Q^2^ intercept was −0.326, −0.338 and −0.174, respectively). Subsequently, heatmaps of metabolic characteristics of liver, serum and intestine content were carried out to view the data more intuitively (Figure 3A–C). The results of heatmaps were consistent with those of PCA and PLS-DA.

### 2.3. Metabolic Alteration Between NAFLD Models and Control Group

**Figure 4:**
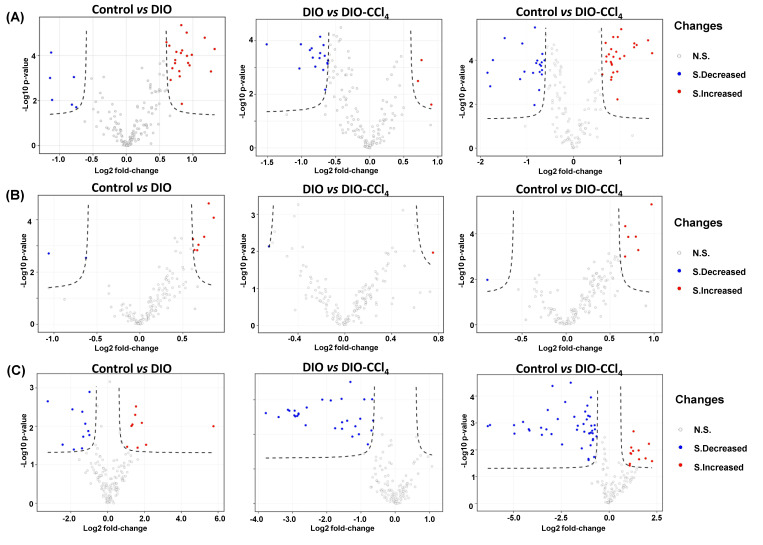
Volcano plots of metabolic features of liver (**A**), serum (**B**) and intestine content (**C**).

As illustrated above, the metabolic characteristics of NAFLD model groups could be distinctly distinguished from those of control group. To further discover the disturbed metabolites, a fold change analysis and t-test of the data were performed, visualizing on a volcano plot (Figure 4). False positive rate (FDR) correction was performed to reduce the FDR based on the Benjamini−Hochberg method [18]. Features with a fold change (FC) > 1.6 or FC < 0.6, *p* < 0.05, and FDR < 0.05 were defined as significantly altered metabolites (Supplementary Tables S2 and S3). A total of 47 metabolites were changed in liver, seven in serum and 56 in intestine content samples. To further analyze the distinction of liver, serum and intestine content samples, Venn analysis was performed (Figure 3D). The results showed that 17 metabolites were shared between liver and intestine content, while only five between liver and serum, and one between serum and intestine content, so the metabolic alteration of liver was more similar to that of intestine than that of serum. Stated thus, we speculated that liver, as the pathological tissue of NAFLD, displayed the most significant alteration of metabolome. Therefore, further analysis was focused on metabolic variation in liver. According to liver data, 19 metabolites were significantly different between DIO and control (Table 1, Table 2 and Table 3). Among them, most were lipids except putrescine. Forty-seven differential metabolites were observed between DIO-CCl_4_ and control (Table 1, Table 2 and Table 3). Twelve metabolites were significantly different between DIO-CCl_4_ and DIO (Table 1, Table 2 and Table 3). Two metabolites, that is PC ae C38:2 and SM C18:1, were detected in all three different comparisons.

### 2.4. Metabolic Pathways and Metabolite Enrichment Analysis Distinguished NASH Models

**Figure 5:**
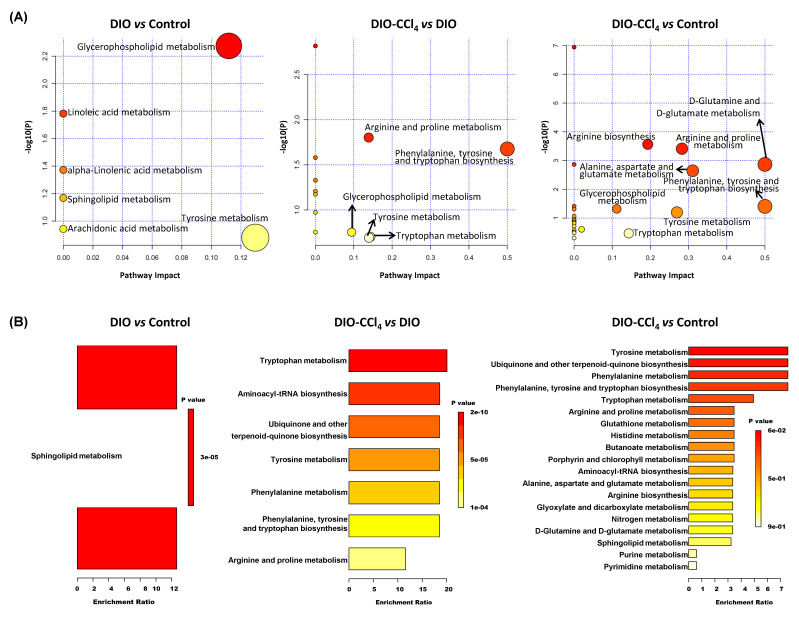
Pathway and enrichment analysis of liver perturbed metabolites. (**A**) Pathway analysis of the differential metabolites. (**B**) Enrichment analysis of the differential metabolites.

To reveal the metabolic processes of the metabolites, pathway analysis and enrichment analysis were conducted using the MetaboAnalyst 4.0. Pathway analysis showed a total of 23 matched pathways. As depicted in Figure 5A, the lipid metabolism, including glycerophospholipid (GPLs) metabolism, linoleic acid metabolism, and alpha-linolenic acid metabolism, was observed to be disturbed in DIO group compared with control group (*p* < 0.05). The perturbation of aminoacyl-tRNA biosynthesis, nitrogen metabolism, lipid metabolism (the same as DIO versus control), glyoxylate and dicarboxylate metabolism, and amino metabolism (including arginine biosynthesis, arginine and proline metabolism, D-glutamine and D-glutamate metabolism, alanine, aspartate and glutamate metabolism, phenylalanine, and tyrosine and tryptophan biosynthesis) were also discovered between DIO-CCl_4_ and control groups (*p* < 0.05). Five pathways were changed between DIO-CCl_4_ and DIO, as most belonging to the amino acid (AA) metabolism.

Moreover, metabolite enrichment analysis was conducted using quantitative metabolite data (Figure 5B). Only sphingolipids (SPs) metabolism was changed (DIO vs. control, *p* < 0.05), while five sets exhibited high significance between DIO-CCl_4_ and control (*p* < 0.05), which corresponded to tyrosine metabolism, ubiquinone and other terpenoid-quinone biosynthesis, phenylalanine metabolism, phenylalanine, tyrosine and tryptophan biosynthesis, and tryptophan metabolism. In addition, the disturbed pathways between DIO-CCl_4_ and DIO were very similar to those between DIO-CCl_4_ and control, indicating that the disturbance of DIO-CCl_4_ was mainly correlated to additional treatment of CCl_4_.

### 2.5. Correlation Analysis between the Differential Metabolites and NAFLD-Associated Parameters

**Figure 6:**
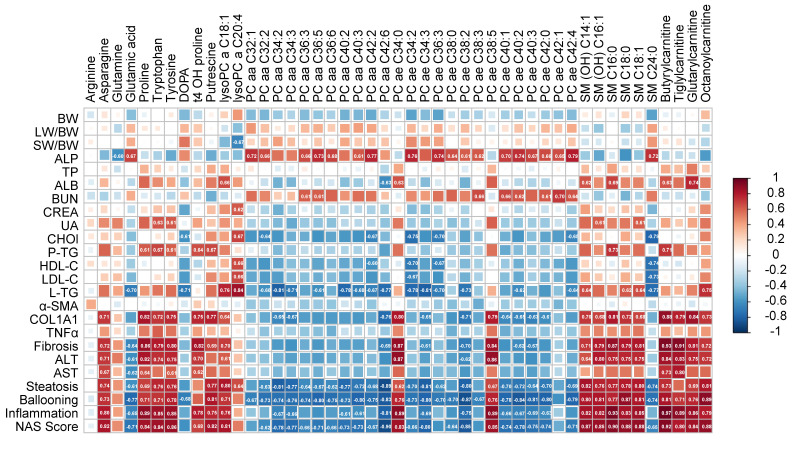
The correlations analysis between disease parameters and liver perturbed metabolites levels.

Correlation analysis was performed to obtain a visualized understanding of the relationship between metabolome and NAFLD-associated parameters (Figure 6, Table S4). Clinical parameters, including body weight (BW), liver/BW ratio, TNF-α and α-SMA, were not associated with the differential metabolites. While ALP, ALB, BUN, serum TG, hepatic TG, Col-1a1 and hallmarks of liver function (such as fibrosis, steatosis, ballooning, inflammation and NAS Score) were significantly correlated to the differential metabolites. Among them, Col-1a1, ALT, AST, hallmarks of liver function, serum TG and hepatic TG shared the same pattern, while ALP and BUN shared the same pattern. Most of the metabolites, except arginine, were correlated to the clinical parameters. The levels of Col-1a1, ALT, AST, hallmarks of liver function were positively associated with the levels of asparagine, proline, tryptophan, tyrosine, t4 OH proline, putrescine, lysoPC a C18:1, several carnitines and sphingomyelins (SM). In contrast, the levels of several phosphtidylcholines (PC) showed the inverse pattern.

### 2.6. Amelioration of Obeticholic Acid (OCA) on NASH

**Figure 7:**
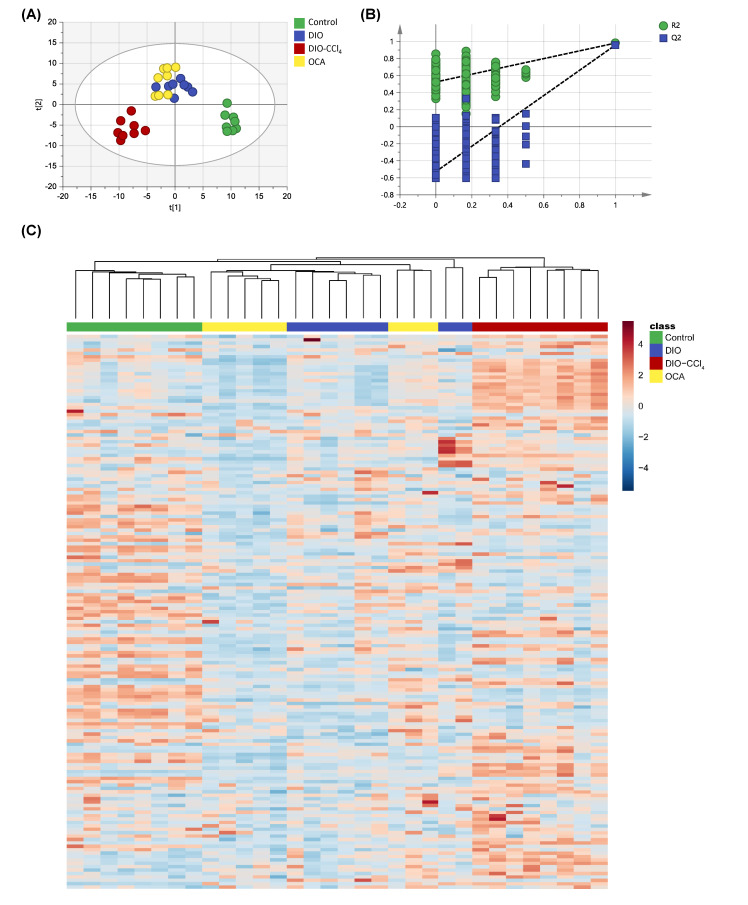
The metabolomic analysis of liver among control, model and OCA treated groups. (**A**) PLS-DA score plot of liver. (**B**) Model validation of PLS-DA using a permutation test with 200 cycles. (**C**) Heatmap of metabolic features of liver.

**Figure 8:**
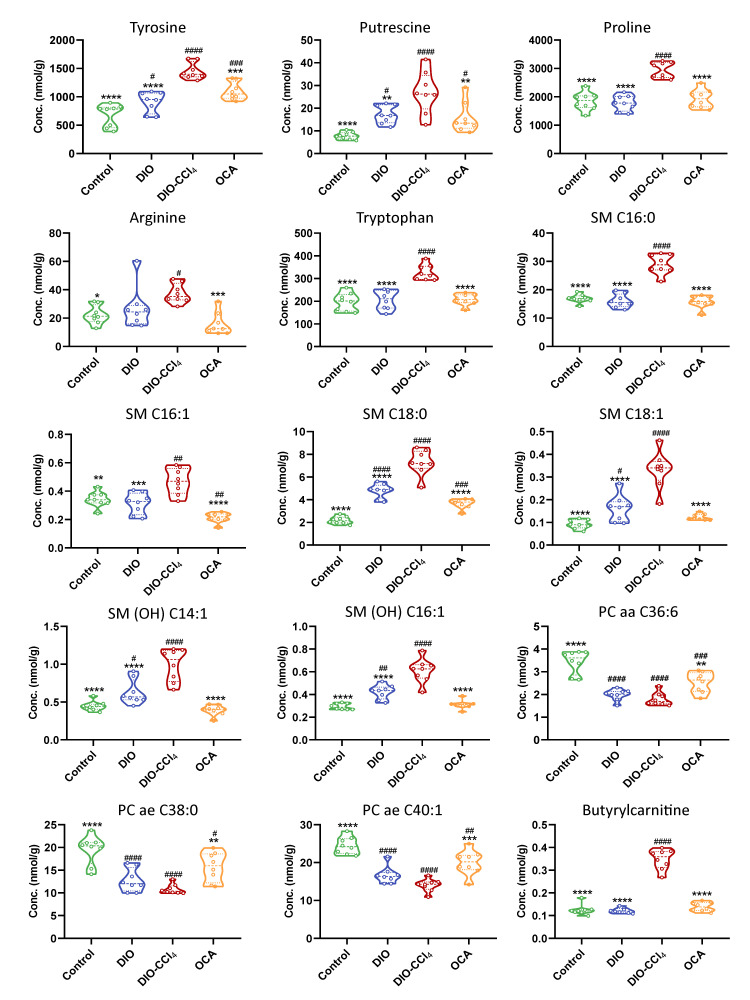
Violin plots of the differential metabolites of liver. * *p* < 0.05, ** *p* < 0.01, *** *p* < 0.005 and **** *p* < 0.001 vs. the DIO-CCl_4_ group; ^#^ *p* < 0.05, ^##^ *p* < 0.01, ^###^ *p* < 0.005 and ^####^ *p* < 0.001 vs. the Control group.

Additionally, we studied effect of OCA, which was a drug candidate for the treatment of NASH. We expected that metabolomics analysis could provide some interpretation to support the therapeutic action of OCA. MSA including PLS-DA and heatmap was performed to understand the effect of OCA more clearly. The PLS-DA result showed that OCA group was distinguished from DIO-CCl_4_ group and grouped with DIO group (Figure 7A). Heatmap indicated the similar result (Figure 7C). These results revealed that OCA might attenuate the injury caused by CCl_4_. Subsequently, the effect of OCA on metabolites was investigated by measuring the levels of differential metabolites obtained by comparing DIO-CCl_4_ group with OCA group (Figure 8). The intervention of OCA had increased PC levels (such as PC aa C36:6, PC ae C38:0 and PC ae C38:0), while it decreased the levels of tyrosine, putrescine, proline, tryptophan, arginine, butyrylcarnitine, SM C16:0, SM C16:1, SM C18:0, SM C18:1, SM (OH) C14:1 and SM (OH) C16:1. Among them, the levels of proline, tryptophan, butyrylcarnitine, SM C16:0, SM C18:1, SM (OH) C14:1 and SM (OH) C16:1 in OCA group trended to be similar to control group, indicating that these metabolites might be contributed to the modulated effect of OCA.

## 3. Discussion

Currently, the prevalence of NAFLD is significantly increasing (from 15% in 2005 to 25% in 2010), while the rate of NASH has almost doubled (59.1% versus 33%) [19,20]. NASH transforms to cirrhosis or HCC more easily than NAFL. However, the mechanism of the transition from NAFL to NASH remains poorly understood. OCA was shown to improve liver histology in patients with NASH in randomized, placebo-controlled clinical trials [21,22]. In present study, we aimed to provide metabolic information which might be helpful to understand mechanism of NAFLD progress and amelioration of OCA on NASH. The results showed that treatment of DIO caused steatosis and ballooning, while inflammation and fibrosis only occurred in co-treatment of DIO and CCl_4_. The levels of ALT and AST, as markers of hepatocyte injury, significantly increased in DIO-CCl_4_ group, speculating that hepatocyte injury was mainly caused by CCl_4_. The PCA and PLS-DA results of control and NAFLD model group showed the obvious separation of each group, suggesting the altered metabolite levels in different conditions. In addition, liver samples showed the best separation, indicating that the greatest metabolic alteration might occur in liver. OCA administration to DIO-CCl_4_ mice showed significantly decrease in serum ALT, AST and TG as well as the hepatic steatosis, inflammation and fibrosis, which was also observed in previous research [23,24,25]. The PLS-DA plot of four groups showed that the treatment of OCA might ameliorate the injury of CCl_4_, according to the close distance of DIO and OCA groups.

Volcano plots were performed to investigate differential metabolites, and metabolic profiling here revealed several metabolic pathways and specific metabolites associated with the development of NAFLD (from control to NAFL following to NASH). Comparing with differential metabolites of liver, serum, and intestine content, liver and intestine content showed more differential metabolites than those of serum. The metabolic changes in serum may reflect response of the whole body to disease [26], and the alteration in pathological liver may be weakened in serum during blood circulation. So the differential metabolites of serum were less than that of liver and intestine content. Moreover, liver and intestine content shared more disturbed metabolites, especially 11 acyl-alkyl-GPLs (the PC ae C group) and two SPs (the SM(OH)C and SM C groups). According to previous literatures, the gut and liver are intrinsically connected and mainly relied on each other based on metabolic functioning [27]. Alteration in the gut–liver axis, including increased gut dysbiosis and permeability, are related to NAFLD [28]. Comparing with the metabolite levels of DIO, DIO-CCl_4_ and control groups, only 19 and 12 differential metabolites were observed between DIO and control, and between DIO and DIO-CCl_4_, while 47, more than the sum of 19 and 12, between DIO-CCl_4_ and control. These results showed that the increasing metabolic disturbance occurred during co-treatment of DIO and CCl_4_.

Pathway and enrichment analysis results suggested that lipid metabolism and AA metabolism were most significantly regulated and associated with NAFLD onset. Among them, SPs metabolism and GPLs metabolism showed significant disturbance comparing with DIO and control group, while AA metabolism enormously altered comparing with DIO and DIO-CCl_4_ groups. The results indicated that HFD might alter lipid metabolism, while CCl_4_ might disturb AA metabolism. In recent lipidomics study of NAFLD, NASH and cirrhosis, a total of 48 lipid species were investigated in liver and plasma [29]. Among them, SPs and GPLs were most predictive of a specific disease stage, and we also found that the levels of SPs and GPLs were disturbed during NAFLD process. Detailed analysis of lipid metabolism showed that the concentrations of PC aa C group (diacyl-GPLs) decreased. Similarly, the levels of PC ae C group was also decreased except for PC ae C34:0 and PC ae C38:5, which is consistent with the previous report and the function of these phosphatidylcholines (PCs) [30]. In contrast, lysophosphatidylcholines (LPCs) and sphingomyelin (SM) was detected in up-regulated sets except for SM C24:0. Correlation analysis between differential metabolites and clinical parameters showed that the levels of PCs, except for PC ae C34:0 and PC ae C38:5, were negatively associated with hepatocyte injury index (ALT and AST) and clinical hallmarks of NASH (fibrosis score, steatosis score, ballooning score, inflammation score, and NAS score). PC and phosphatidylethanolamine (PE) are major phospholipids in mammalian membranes. Previous literatures reported that hepatic PC/PE ratio displayed an important clinical significance [31]. Lots of patients with NAFLD and NASH have a lower hepatic PC/PE ratio than that of healthy subjects [32]. In our study, the levels of PE nearly unchanged, while PC significantly decreased. So hepatic PC/PE ratio decreased which was consistent with the literature. A decreased PC/PE ratio could adversely affect membrane integrity, leading to liver injury [30]. In addition, a decreased PC/PE ratio might initiate inflammation, because the flow of ions (such as calcium) and ion radicals varies in membrane potential, and C-reactive protein, as inflammatory response protein, bound more avidly to cell membranes. However, the levels of LPCs and SMs, except for SM C24:0, showed reverse correlated pattern of PCs. Previous work indicated that increasing levels of SMs were observed in liver and serum in HFD-fed mice [33,34], because SM reduction ameliorates both insulin sensitivity and steatosis [34,35]. Relevantly, metabolomic analysis of human serum suggested that SMs might be up-regulated in NASH patients [36].

In addition, AA metabolism was also significantly altered among different groups. These results were not surprising, because liver is the site of protein and AA metabolism. Eight AAs (arginine, asparagine, glutamine, proline, tryptophan, tyrosine, t4 OH proline, putrescine) were up-regulated, with glutamic acid and DOPA being down-regulated. Pathway analysis showed that phenylalanine, tyrosine and tryptophan biosynthesis, D-glutamine and D-glutamate metabolism, and arginine and proline metabolism were significantly disturbed. Recent metabolomics-based studies suggested that AA metabolism plays an important role in NAFLD pathogenesis [37], and aromatic AAs (AAAs, such as tyrosine and phenylalanine) were also found altered with increased severity of liver diseases [38]. Nonessential amino acids (serine, glycine, glutamate, glutamine, aspartate, asparagine, and alanine) and essential amino acids (valine and methionine) were also reported that involved in the appearance of NASH. In addition, acylcarnitines (such as butyrylcarnitine, tiglylcarnitine, glutarylcarnitine, and octanoylcarnitine) were also affected, and were positively associated with hepatocyte injury index and clinical hallmarks of NASH. And alterations of carnitines were reported to result in high mitochondrial lipotoxicity, lipid loads, and altered lipid metabolism [39].

Moreover, the concentrations of differential metabolites in control, DIO, DIO-CCl_4_ and OCA groups were analyzed to discover whether the treatment of OCA could modulate the altered metabolites. As shown in Figure 8, OCA could attenuate the levels of several SMs, AAs (proline, tryptophan, tyrosine, arginine, and putrescine) and butyrylcarnitine and increase the level of several PCs, while no significant changes were observed in LPCs. AAs could alleviate hepatic steatosis and liver injury associated with NASH by suppressing FAS gene expression and protein levels [40]. PC directly contributes to hepatic TG synthesis and thus may promote the development of steatosis [41]. Combining with the histological and metabolic characteristics, steatosis score and hepatic TG significantly decreased along with the changed level of AAs and PCs, which indicated that OCA might reverse hepatic steatosis throughout AA metabolism, and PC biosynthesis and lipoprotein metabolism. LPC was generated from the hydrolysis of PC by the action of phospholipase A_2_, and were associated with cell injury and apoptosis in NASH, and could cause membrane toxicity and promote inflammation [42]. OCA could not decrease the level of LPCs, indicating that OCA could not decrease membrane toxicity and cell injury.

In present study, we induced the mice progressed from NAFL to NASH by fedding the mice with high-fat diet (60% fat calories) for 6 weeks and then intraperitoneally injected with CCl_4_ and continued the high-fat diet for 4 weeks. It is an effective mouse model for initial in vivo proof-of-concept studies on antisteatosis and antifibrotic therapies in a relative short time. However, this model dose not closely mirror the etiology and natural history of NASH, which progressed slowly and might take decades in human. The traditional Western diet—which is high in fat, fructose and cholesterol would promote hepatic insulin resistance, marked steatohepatitis, inflammation and perisinusoidal fibrosis in mice after fed for 26 weeks. Further research might be conducted in the Western diet—induced mice in the future to investigate the correlation among metabolites, histological and metabolic characteristics in the pathogenesis of NASH.

## 4. Materials and Methods

### 4.1. Chemicals

Ultrapure water was prepared by a Milli-Q water purification system (Millipore, Molsheim, France). HPLC grade acetonitrile, ethanol, isopropanol and methanol were obtained from Merck (Darmstadt, Germany). Formic acid was purchased from Merck. Ammonium bicarbonate, pyridine and phosphate buffered saline (PBS) were purchased from Sigma-Aldrich (St. Louis, MO, USA). Carbon tetrachloride (CCl_4_) and CMC-Na were purchased from Aladdin (Shanghai, China).

### 4.2. Ethics Statement and Animals

C57Bl/6J male mice were acquired from Shanghai Lingchang Laboratory Animal Care Co., Ltd. (Shanghai, China). The mice were housed in a controlled environment (12 h light/dark cycle, 22–24 °C, humidity 50% ± 10%). All animal procedures were approved by the Institutional Animal Care and Utilization Committee (IACUC) (Shanghai, China) of Shanghai Institute of Materia Medica (SIMM), Chinese Academy of Sciences (CAS) (Shanghai, China), with accreditation number of 2017-12-LY-72. Mice were fed ad libitum with a high-fat diet (60 kcal% fat, 20 kcal% protein and 20 kcal% carbohydrate; Cat. D12492i, Research Diet, New Brunswick, NJ, USA) for 10 weeks before and during the experiment. A corresponding group of C57Bl/6J male mice (*n* = 8) fed with standard diet was set as control group. After 6 weeks, HFD mice were stratified divided into three groups (*n* = 8) based on their body weights and treated as follows: (i) DIO group, in which mice were orally administrated with vehicle (0.25% CMC-Na) once daily for 4 weeks; (ii) DIO-CCl_4_ group, in which mice were intraperitoneally injected with CCl_4_ 0.05 mL/kg twice a week and orally administrated with vehicle once daily for 4 weeks; and (iii) OCA group, in which mice were intraperitoneally injected with CCl_4_ 0.05 mL/kg twice a week and orally administered with OCA (30 mg/kg) once daily for 4 weeks. At the end of treatment, animals were anesthetized and sacrificed according to institutional guidelines. From each animal, serum, liver and intestine content were collected.

### 4.3. Biochemical Analysis, Histology and NAFLD Activity Score and Fibrosis Evaluation

Serum alanine aminotransferase (ALT) and aspartate aminotransferase (AST) were measured using an Infinity Enzymatic Assay Kits (Shino-test Corporation, Tokyo, Japan). Serum triglyceride (TG) and cholesterol (TC) were determined using commercial kits (DongOu Jin Ma Biotech, Wenzhou, China). For histology, the harvested liver tissues from all study groups were fixed in 10% buffered formalin. Then the paraffin embedding, sectioning and HE staining of liver tissue were commissioned to Shanghai Tianhe Biotechnology Co., Ltd. (Shanghai, China). The images were reviewed and analyzed under microscope (Olympus 1X73, Olympus Corporation, Tokyo, Japan) at 200× magnification. The NAFLD activity score (NAS) was calculated from the sum of the individual scores for steatosis, inflammation and ballooning according to the NASH CRN scoring system [43,44]. The formalin-fixed, paraffin-embedded liver sections were stained with Sirius Red (Nanjing SenBeiJia Biological Technology Co., Ltd., Nanjing, China) for assessment of fibrosis, and the complete scan was performed using Nano Zoomer 2.0HT (Hamamatsu Photonics K.K., Hamamatsu, Japan) and Image Pro Plus software (Media Cybernetics, Inc., Rockville, MD, USA) was used to calculate the positive area ratio of Sirius red. Hepatic TG was detected using a heptane-isopropanol-Tween mixture (3:2:0.01 by volume) and measured by a commercial kit and normalized by tissue weight.

### 4.4. RNA Isolation and Real-Time PCR

Total RNA was extracted from liver tissues using TRIzol reagent (Life Technologies, Carlsbad, CA, USA). cDNA was generated by a Primer Script RT reagent kit (TaKaRa Biotechnology, Dalian, China) and analyzed via quantitative PCR. All of the primer sequences used in this study are included in Table S1. The relative amount of individual mRNA was normalized to β-actin mRNA.

### 4.5. Metabolomics

The AbsoluteIDQ^®^ p180 Kit (Biocrates Life Sciences, Innsbruck, Austria) was applied to assay a total of 188 metabolites from five analyte groups, including acylcarnitines, amino acids (AA), biogenic amines, glycerophospho- and sphingolipids, and hexose. The workflow was followed to the AbsoluteIDQ^®^ p180 Kit manufacturer instructions. The serum samples were centrifuged at 4 °C for 5 min at 2750× *g*. After homogenizing the liver and intestine content samples with water (1:5, g/mL), the suspensions were centrifuged at 3220× *g* for 10 min at 4 °C. A 10-μL of the Biocrates internal standard solution was added into each well of the 96-well extraction plate. And then, 10 μL of serum, liver and intestine content samples, quality control (QC) samples, blank, zero samples (PBS), or calibration standards was accurately pipetted to the appropriate wells. The LC-MS/MS and FIA-MS/MS analysis were performed using an ACQUITY UPLC system (H-Class, Waters, Milford, MA, USA) coupled with a Triple Quad 5500 (AB SCIEX, Concord, Canada) mass spectrometer equipped with an electrospray ionization (ESI) source. Separation chromatographic column was Waters BEH C18 UPLC column (2.1 × 75 mm, 1.7 μm) (Waters, Milford, MA, USA). Multiple reaction monitoring (MRM) transitions for each analyte and internal standard were collected over a scheduled retention time window using tune files and acquisition methods from the AbsoluteIDQ^®^ p180 Kit. The median value of all zero samples on the plate was calculated as an approximation of background noise, i.e., as the limit of detection (LOD). Results from the LC-MS/MS assay together with the FIA assay were imported in to MetIDQ (Biocrates Life Sciences, Innsbruck, Austria) for further data evaluation.

### 4.6. Statistical Analysis

All values were presented as the mean ± SEM, and analyzed using Student’s t-test and one-way analysis of variance (ANOVA) using GraphPad Prism 8.0 (GraphPad Software, LaJolla, CA). P value less than 0.05 was considered as statistical significance. Principal component analysis (PCA) and partial least squares-discriminant analysis (PLS-DA) with unit variance (UV) scaling was performed by SIMCA 14.1software (Umetrics, Umea, Sweden). The PLS-DA validation was performed by a permutation test with 200 cycles. The correlation analysis was constructed by SPSS 18.0 and visualized using the R package (corrplot). Pathway analysis and enrichment analysis was performed to identify the disturbed pathways. Univariate Analysis, cluster Analysis, enrichment analysis and pathway analysis, were conducted on the MetaboAnalyst website (http://www.metaboanalyst.ca, accessed on 30 May 2021) [45,46] and iMetaShiny Apps (https://shiny.imetalab.ca, accessed on 30 May 2021) [47].

## 5. Conclusions

In summary, a comprehensive overview of metabolic characteristics and pathway disturbance of NAFLD model groups and control group, along with amelioration of OCA on the treatment of NASH, was provided in this study. Aminoacyl-tRNA biosynthesis, nitrogen metabolism, lipid metabolism, glyoxylate and dicarboxylate metabolism, and amino metabolism were disturbed during progression from control to NAFL and from NAFL to NASH. Additionally, differential metabolites among NAFLD groups and control group were discovered. Among them, metabolic alteration of liver and intestine content was more significant. And several differential metabolites were correlated to some clinical parameters. OCA showed modulated effect according to metabolomics analysis and histological characteristics. To sum up, our research could provide metabolic clues to understand the mechanism of the treatment of OCA.

## Figures and Tables

**Table 1:** Summary of differential metabolites in liver between different groups (amino acids).

	DIO vs. Control			DIO−CCl_4_ vs. DIO			DIO−CCl_4_ vs. Control		
Metabolite	Log2 FC	*p* Value	Change	Log2 FC	*p* Value	Change	Log2 FC	*p* Value	Change
arginine	0.34	0.32	-	0.40	0.21	-	0.74	0.002	↑
asparagine	0.20	0.20	-	0.53	0.00	-	0.73	0.0003	↑
glutamine	0.45	0.14	-	0.39	0.28	-	0.84	0.011	↑
glutamic acid	−0.44	0.02	-	−0.47	0.06	-	−0.92	0.00004	↓
proline	−0.06	0.76	-	0.73	0.0001	↑	0.67	0.0001	↑
tryptophan	0.06	0.68	-	0.66	0.0004	↑	0.73	0.0002	↑
tyrosine	0.42	0.03	-	0.68	0.0001	↑	1.10	0.00002	↑
DOPA	−0.66	0.05	↓	−0.28	0.28	-	−0.94	0.006	↓
t4 OH proline	0.00	0.98	-	0.68	0.001	↑	0.68	0.0001	↑
putrescine	1.14	0.001	↑	0.65	0.03	-	1.79	0.002	↑

“-”: no significantly changed; “↓”: significantly decreased; “↑”: significantly increased.

**Table 2:** Summary of differential metabolites in liver between different groups (PCs and LPCs).

	DIO vs. Control			DIO−CCl_4_ vs. DIO			DIO−CCl_4_ vs. Control		
Metabolite	Log2 FC	*p* Value	Change	Log2 FC	*p* Value	Change	Log2 FC	*p* Value	Change
lysoPC a C18:1	0.27	0.18	-	0.43	0.02	-	0.70	0.0002	↑
lysoPC a C20:4	0.62	0.09	-	0.05	0.87	-	0.68	0.0003	↑
PC aa C32:1	−0.95	0.0003	↓	0.14	0.16	-	−0.81	0.001	↓
PC aa C32:2	−0.80	0.001	↓	0.00	1.00	-	−0.81	0.001	↓
PC aa C34:2	−0.38	0.02	-	−0.33	0.09	-	−0.70	0.0001	↓
PC aa C34:3	−0.45	0.02	-	−0.37	0.06	-	−0.82	0.0002	↓
PC aa C36:3	−0.69	0.0004	↓	−0.12	0.20	-	−0.82	0.0001	↓
PC aa C36:5	−1.33	0.0001	↓	−0.36	0.00	-	−1.69	0.00005	↓
PC aa C36:6	−0.80	0.0005	↓	−0.14	0.21	-	−0.94	0.0003	↓
PC aa C40:2	−0.50	0.003	-	−0.20	0.09	-	−0.70	0.0001	↓
PC aa C40:3	−0.98	0.0001	↓	−0.32	0.03	-	−1.30	0.00003	↓
PC aa C42:2	−0.83	0.000005	↓	−0.03	0.63	-	−0.85	0.00001	↓
PC aa C42:6	−0.38	0.02	-	−0.71	0.003	↓	−1.09	0.0001	↓
PC ae C34:0	0.03	0.64	-	0.60	0.001	-	0.62	0.0001	↑
PC ae C34:2	−0.91	0.0001	↓	−0.04	0.75	-	−0.95	0.00001	↓
PC ae C34:3	−0.51	0.004	-	−0.34	0.05	-	−0.85	0.0001	↓
PC ae C36:3	−0.82	0.0001	↓	0.06	0.67	-	−0.76	0.0001	↓
PC ae C38:0	−0.61	0.003	-	−0.24	0.09	-	−0.85	0.0004	↓
PC ae C38:2	−0.84	0.0001	↓	−0.76	0.001	↓	−1.60	0.00001	↓
PC ae C38:3	−0.67	0.001	↓	−0.13	0.32	-	−0.80	0.0001	↓
PC ae C38:5	0.07	0.36	-	0.80	0.001	↑	0.87	0.0004	↑
PC ae C40:1	−0.56	0.00	-	−0.25	0.02	-	−0.81	0.00003	↓
PC ae C40:2	−0.91	0.00001	↓	−0.40	0.001	-	−1.30	0.00002	↓
PC ae C40:3	−0.65	0.00004	↓	−0.38	0.01	-	−1.02	0.000004	↓
PC ae C42:0	−0.73	0.0002	↓	−0.26	0.003	-	−0.99	0.0001	↓
PC ae C42:1	−0.73	0.0002	↓	0.12	0.41	-	−0.62	0.0004	↓
PC ae C42:4	−1.18	0.00002	↓	−0.18	0.04	-	−1.35	0.00002	↓

“-”: no significantly changed; “↓”: significantly decreased; “↑”: significantly increased.

**Table 3:** Summary of differential metabolites in liver between different groups (SMs and acylcarnitines).

	DIO vs. Control			DIO−CCl_4_ vs. DIO			DIO−CCl_4_ vs. Control		
Metabolite	Log2 FC	*p* Value	Change	Log2 FC	*p* Value	Change	Log2 FC	*p* Value	Change
SM OH C14:1	0.49	0.02	-	0.65	0.01	↑	1.15	0.001	↑
SM OH C16:1	0.52	0.001	-	0.53	0.002	-	1.06	0.0003	↑
SM C16:0	−0.07	0.52	-	0.85	0.0002	↑	0.78	0.0001	↑
SM C18:0	1.13	0.0001	↑	0.60	0.001	-	1.73	0.0001	↑
SM C18:1	0.82	0.02	↑	1.03	0.001	↑	1.85	0.0004	↑
SM C24:0	−0.75	0.0001	↓	0.06	0.74	-	−0.69	0.00002	↓
Butyrylcarnitine	−0.03	0.76	-	1.51	0.0001	↑	1.48	0.00001	↑
Tiglylcarnitine	−0.05	0.91	-	0.88	0.0002	↑	0.83	0.000003	↓
Glutarylcarnitine	0.07	0.63	-	0.61	0.001	-	0.68	0.0004	↓
Octanoylcarnitine	0.46	0.01	-	0.34	0.02	-	0.80	0.0001	↓

“-”: no significantly changed; “↓”: significantly decreased; “↑”: significantly increased.

## Data Availability

Data used in this study is available on the request from the corresponding author.

## Supplementary Materials

The following are available online at https://www.mdpi.com/article/10.3390/metabo11060374/s1, Table S1: Primer sequences used for real-time PCR of mouse liver. Table S2: Summary of differential metabolites in serum between different groups. Table S3: Summary of differential metabolites in intestine content between different groups. Table S4: Biochemical parameters of different groups. Table S5: Body weights prior to the onset of treatment (CCl_4_).
