# Supplementary material for: Metabolomic Study of High-Fat Diet-Induced Obese (DIO) and DIO Plus CCl4-Induced NASH Mice and the Effect of Obeticholic Acid
Source: Metabolites. 2021 Jun 10;11(6):374. doi: 10.3390/metabo11060374 (PMC8230384; doi:10.3390/metabo11060374)
Supplement: Supplementary file 1 [file metabolites-11-00374-s001.zip › metabolites-1233466-supplementary.pdf]

Table S1. Primer sequences used for real-time PCR of mouse liver tissues.

Genes	Forward sequences (5′ to 3′)	Reverse sequences (5′ to 3′)
**Col-1a1**	ACCTGTGTGTTCCCTACTCA	GACTGTTGCCTTCGCCTCTG
**MCP-1**	GCAGCCTGAAGAGTGGTACTCTC	CATTCAGCCAACATTCCCATCTC
**Cyclin D1**	GCACAACGCACTTTCTTTCCA	CGCAGGCTTGACTCCAGAAG
**CDK1**	AGAAGGTACTTACGGTGTGGT	GAGAGATTTCCCGAATTGCAGT
**CDK2**	CCTGCTCATTAATGCAGAGGG	GTGCTGGGTACACACTAGGTG
**CDK4**	ATGGCTGCCACTCGATATGAA	TCCTCCATTAGGAACTCTCACAC
**β-actin**	GGCTGTATTCCCCTCCATCG	CCAGTTGGTAACAATGCCATGT

Table S2. Summary of differential metabolites in serum between different groups.

	DIO vs Control			DIO–CCl_4 vs DIO			DIO–CCl_4 vs Control		
**Metabolite**	**Log2 FC**	**p value**	**Change**	**Log2 FC**	**p value**	**Change**	**Log2 FC**	**p value**	**Change**
Glutamic.acid	−1.06	0.002	↓	0.17	0.35	-	−0.89	0.01	↓
Carnosine	0.67	0.001	↑	0.15	0.13	-	0.82	0.001	↑
PC.aa.C34.2	0.31	0.02	-	0.37	0.01	-	0.67	0.00005	↑
PC.aa.C36.2	0.47	0.002	-	0.24	0.09	-	0.71	0.0001	↑
PC.aa.C42.6	0.86	0.0001	↑	−0.06	0.63	-	0.79	0.0001	↑
PC.ae.C38.2	0.80	0.00002	↑	0.17	0.11	-	0.97	0.00001	↑
PC.ae.C40.3	0.63	0.001	-	0.04	0.70	-	0.67	0.001	↑

Table S3. Summary of differential metabolites in intestine content between different groups.

	DIO vs Normal			DIO–CCl_4 vs DIO			DIO–CCl_4 vs Normal		
**Metabolite**	**Log2 FC**	**p value**	**Change**	**Log2 FC**	**p value**	**Change**	**Log2 FC**	**p value**	**Change**
citrulline	1.15	0.05	-	0.15	0.88	-	1.30	0.002	↑
glycine	0.43	0.21	-	0.57	0.14	-	1.01	0.001	↑
Isoleucine	0.61	0.16	-	0.49	0.24	-	1.10	0.001	↑
leucine	0.51	0.22	-	0.38	0.40	-	0.89	0.002	↑
lysine	0.60	0.16	-	0.54	0.22	-	1.14	0.0002	↑
methionine	0.72	0.16	↑	0.25	0.68	↑	0.97	0.01	↑
phenylalanine	0.53	0.22	-	0.42	0.40	-	0.96	0.002	↑
serine	0.65	0.09	↑	0.60	0.10	-	1.25	0.0004	↑
threonine	0.32	0.36	-	0.54	0.22	-	0.86	0.002	↑
tryptophan	1.10	0.14	↑	0.24	0.82	-	1.34	0.001	↑
tyrosine	0.54	0.24	-	0.30	0.63	-	0.84	0.004	↑
valine	0.77	0.13	↑	0.41	0.43	-	1.17	0.001	↑
methionine.sulfoxide	0.44	0.19	-	0.30	0.43	-	0.74	0.002	↑
lysoPC.a.C14.0	0.55	0.003	-	0.40	0.003	-	0.95	0.0001	↑
lysoPC.a.C16.0	0.91	0.09	↑	0.91	0.04	↑	1.82	0.001	↑
lysoPC.a.C17.0	−0.28	0.88	-	1.06	0.01	↑	0.79	0.02	↑
lysoPC.a.C18.0	−0.07	0.72	-	1.76	0.01	↑	1.68	0.002	↑
lysoPC.a.C18.1	0.24	0.30	-	0.42	0.05	-	0.66	0.003	↑
lysoPC.a.C20.4	1.06	0.01	↑	−0.02	0.88	-	1.03	0.002	↑
lysoPC.a.C24.0	−1.49	0.01	↓	0.38	0.01	-	−1.12	0.01	↓
lysoPC.a.C26.0	−1.54	0.003	↓	0.38	0.11	-	−1.16	0.01	↓
lysoPC.a.C26.1	−1.29	0.01	↓	0.14	0.43	-	−1.15	0.01	↓
PC.aa.C28.1	0.66	0.03	↑	2.86	0.003	↑	3.52	0.003	↑
PC.aa.C30.0	0.56	0.04	-	2.13	0.001	↑	2.69	0.001	↑
PC.aa.C32.0	0.85	0.11	↑	1.16	0.01	↑	2.01	0.00003	↑
PC.aa.C34.4	−1.34	0.01	↓	0.09	0.69	-	−1.25	0.01	↓
PC.aa.C36.1	−0.48	0.39	-	1.55	0.004	↑	1.08	0.02	↑
PC.aa.C36.5	−1.86	0.01	↓	−0.22	0.22	-	−2.08	0.01	↓
PC.aa.C38.0	0.41	0.06	-	0.44	0.08	-	0.85	0.004	↑
PC.aa.C40.4	−0.13	0.84	-	−0.96	0.04	↓	−1.08	0.03	↓
PC.ae.C30.0	−0.14	0.73	-	0.90	0.001	↑	0.76	0.001	↑
PC.ae.C34.3	−2.08	0.03	↓	−0.16	0.63	-	−2.24	0.03	↓
PC.ae.C36.0	0.24	0.12	-	1.40	0.004	↑	1.64	0.001	↑
PC.ae.C36.1	−0.12	0.68	-	1.55	0.01	↑	1.43	0.01	↑
PC.ae.C38.0	1.00	0.02	↑	−0.06	0.97	-	0.93	0.001	↑
PC.ae.C38.2	−0.90	0.09	↓	−0.67	0.14	↓	−1.57	0.02	↓
PC.ae.C40.1	−0.47	0.22	-	−1.07	0.08	↓	−1.54	0.01	↓

PC.ae.C40.2	−0.17	0.72	-	1.25	0.01	↑	1.08	0.02	↑
PC.ae.C40.4	−0.74	0.02	↓	−0.54	0.07	-	−1.27	0.002	↓
PC.ae.C40.5	−1.26	0.06	↓	−0.64	0.05	-	−1.90	0.02	↓
PC.ae.C42.3	−0.73	0.11	↓	−0.34	0.27	-	−1.08	0.04	↓
PC.ae.C42.4	−5.75	0.01	↓	−0.30	0.66	-	−5.75	0.01	↓
SM..OH..C14.1	1.22	0.01	↑	2.95	0.003	↑	4.17	0.002	↑
SM..OH..C16.1	0.46	0.10	-	2.83	0.002	↑	3.28	0.002	↑
SM..OH..C22.1	3.26	0.002	↑	3.09	0.002	↑	6.35	0.001	↑
SM..OH..C22.2	1.55	0.07	↑	3.41	0.003	↑	4.95	0.002	↑
SM..OH..C24.1	1.33	0.02	↑	1.88	0.001	↑	3.21	0.0003	↑
SM.C16.0	0.62	0.05	-	2.94	0.002	↑	3.56	0.002	↑
SM.C16.1	1.84	0.04	↑	3.14	0.002	↑	4.98	0.001	↑
SM.C18.0	1.38	0.004	↑	2.82	0.003	↑	4.20	0.002	↑
SM.C18.1	0.10	0.67	-	2.92	0.003	↑	3.02	0.003	↑
SM.C24.0	1.92	0.004	↑	2.59	0.002	↑	4.50	0.001	↑
SM.C24.1	2.45	0.03	↑	3.78	0.002	↑	6.23	0.001	↑
SM.C26.0	1.40	0.04	↑	1.57	0.001	↑	2.97	0.00004	↑
SM.C26.1	−0.16	0.64	-	2.62	0.01	↑	2.46	0.01	↑
C18	0.99	0.001	↑	1.31	0.0003	↑	2.30	0.0002	↑
C18.1	1.20	0.16	↑	−0.15	0.75	-	1.04	0.002	↑
citrulline	1.15	0.05	↑	0.15	0.88	-	1.30	0.002	↑
glycine	0.43	0.21	-	0.57	0.14	-	1.01	0.001	↑
isoleucine	0.61	0.16	-	0.49	0.24	-	1.10	0.001	↑
leucine	0.51	0.22	-	0.38	0.40	-	0.89	0.002	↑
lysine	0.60	0.16	-	0.54	0.22	-	1.14	0.0002	↑
methionine	0.72	0.16	↑	0.25	0.68	-	0.97	0.01	↑
phenylalanine	0.53	0.22	-	0.42	0.40	-	0.96	0.002	↑
serine	0.65	0.09	↑	0.60	0.10	-	1.25	0.0004	↑
threonine	0.32	0.36	-	0.54	0.22	-	0.86	0.002	↑
tryptophan	1.10	0.14	↑	0.24	0.82	-	1.34	0.001	↑
tyrosine	0.54	0.24	-	0.30	0.63	-	0.84	0.004	↑
valine	0.77	0.13	↑	0.41	0.43	-	1.17	0.001	↑
methionine.sulfoxide	0.44	0.19	-	0.30	0.43	-	0.74	0.002	↑
lysoPC.a.C14.0	0.55	0.003	-	0.40	0.003	-	0.95	0.0001	↑
lysoPC.a.C16.0	0.91	0.09	↑	0.91	0.04	↑	1.82	0.001	↑
lysoPC.a.C17.0	−0.28	0.88	-	1.06	0.01	↑	0.79	0.02	↑
lysoPC.a.C18.0	−0.07	0.72	-	1.76	0.01	↑	1.68	0.002	↑
lysoPC.a.C18.1	0.24	0.30	-	0.42	0.05	-	0.66	0.003	↑
lysoPC.a.C20.4	1.06	0.01	↑	−0.02	0.88	-	1.03	0.002	↑
lysoPC.a.C24.0	−1.49	0.01	↓	0.38	0.01	-	−1.12	0.01	↓
lysoPC.a.C26.0	−1.54	0.003	↓	0.38	0.11	-	−1.16	0.01	↓
lysoPC.a.C26.1	−1.29	0.01	↓	0.14	0.43	-	−1.15	0.01	↓
PC.aa.C28.1	0.66	0.03	↑	2.86	0.003	↑	3.52	0.003	↑
PC.aa.C30.0	0.56	0.04	-	2.13	0.001	↑	2.69	0.001	↑
PC.aa.C32.0	0.85	0.11	↑	1.16	0.01	↑	2.01	0.00003	↑
PC.aa.C34.4	−1.34	0.01	↓	0.09	0.69	-	−1.25	0.01	↓
PC.aa.C36.1	−0.48	0.39	-	1.55	0.004	↑	1.08	0.02	↑
PC.aa.C36.5	−1.86	0.01	↓	−0.22	0.22	-	−2.08	0.01	↓
PC.aa.C38.0	0.41	0.06	-	0.44	0.08	-	0.85	0.004	↑
PC.aa.C40.4	−0.13	0.84	-	−0.96	0.04	↓	−1.08	0.03	↓
PC.ae.C30.0	−0.14	0.73	-	0.90	0.001	↑	0.76	0.001	↑
PC.ae.C34.3	−2.08	0.03	↓	−0.16	0.63	-	−2.24	0.03	↓
PC.ae.C36.0	0.24	0.12	-	1.40	0.004	↑	1.64	0.001	↑
PC.ae.C36.1	−0.12	0.68	-	1.55	0.01	↑	1.43	0.01	↑
PC.ae.C38.0	1.00	0.02	↑	−0.06	0.97	-	0.93	0.001	↑
PC.ae.C38.2	−0.90	0.09	↑	−0.67	0.14	↓	−1.57	0.02	↓
PC.ae.C40.1	−0.47	0.22	–	−1.07	0.08	↓	−1.54	0.01	↓
PC.ae.C40.2	−0.17	0.72	-	1.25	0.01	↑	1.08	0.02	↑
PC.ae.C40.4	−0.74	0.02	↓	−0.54	0.07	-	−1.27	0.002	↓
PC.ae.C40.5	−1.26	0.06	↓	−0.64	0.05	-	−1.90	0.02	↓
PC.ae.C42.3	−0.73	0.11	↓	−0.34	0.27	-	−1.08	0.04	↓
PC.ae.C42.4	−5.75	0.01	↓	−0.30	0.66	-	−5.75	0.01	↓
SM..OH..C14.1	1.22	0.01	↑	2.95	0.003	↑	4.17	0.002	↑
SM..OH..C16.1	0.46	0.10	-	2.83	0.002	↑	3.28	0.002	↑
SM..OH..C22.1	3.26	0.002	↑	3.09	0.002	↑	6.35	0.001	↑
SM..OH..C22.2	1.55	0.07	↑	3.41	0.003	↑	4.95	0.002	↑

SM..OH..C24.1	1.33	0.02	↑	1.88	0.001	↑	3.21	0.0003	↑
SM.C16.0	0.62	0.05	-	2.94	0.002	↑	3.56	0.002	↑
SM.C16.1	1.84	0.04	↑	3.14	0.002	↑	4.98	0.001	↑
SM.C18.0	1.38	0.004	↑	2.82	0.003	↑	4.20	0.002	↑
SM.C18.1	0.10	0.67	-	2.92	0.003	↑	3.02	0.003	↑
SM.C24.0	1.92	0.004	↑	2.59	0.002	↑	4.50	0.001	↑
SM.C24.1	2.45	0.03	↑	3.78	0.002	↑	6.23	0.001	↑
SM.C26.0	1.40	0.04	↑	1.57	0.001	↑	2.97	0.00004	↑
SM.C26.1	−0.16	0.64	-	2.62	0.01	↑	2.46	0.01	↑
C18	0.99	0.001	↑	1.31	0.0003	↑	2.30	0.0002	↑
C18.1	1.20	0.16	↑	−0.15	0.75	-	1.04	0.002	↑

Table S4. Biochemical parameters of different groups.

	**Normal**		**DIO**		**DIO−CCl_4**		**OCA**	
	**Mean**	**SEM**	**Mean**	**SEM**	**Mean**	**SEM**	**Mean**	**SEM**
**ALP (U/L)**	156.8	6.97	112.9	4.19	121.9	3.08	116.1	3.70
**TP (g/L)**	46.7	0.68	45.6	0.71	47.8	0.8	48.1	0.73
**ALB (g/L)**	15.8	0.35	15.4	0.3	17.1	0.33	17.1	0.23
**BUN (mmol/L)**	10.6	0.24	9.00	0.36	9.43	0.32	9.48	0.41
**CREA (umol/L)**	10.8	0.67	12.4	0.69	12.1	0.45	9.81	0.46
**UA (umol/L)**	59.5	6.05	66.4	5.72	81.8	9.43	69.1	10.9
**CHOl (mmol/l)**	1.97	0.07	3.17	0.17	2.54	0.14	2.34	0.06
**Serum TG (mmol/L)**	0.32	0.03	0.25	0.01	0.61	0.09	0.34	0.03
**Liver TG (umol/g)**	21.5	1.8	41.6	7.3	46.6	3.2	13.4	1.3
**HDL−C (mmol/L)**	1.55	0.06	2.43	0.12	1.84	0.07	1.87	0.04
**LDL−C (mmol/L)**	0.17	0.01	0.36	0.04	0.27	0.04	0.28	0.07

Table S5. Body weights prior to the onset of treatment (CCl_4) .

	**Control**	**DIO**	**DIO−CCl_4**	**OCA**
Pre−treatment (g)	26.3 ± 0.4	31.0 ± 0.8**	30.9 ± 0.4	30.7 ± 0.5

Results are mean ± SE (*n* = 8). ***p*<0.01 versus normal mice.
